# Supplementary material for: Engineering Multicolor Radiative Centers in hBN Flakes by Varying the Electron Beam Irradiation Parameters
Source: Nanomaterials (Basel). 2023 Feb 15;13(4):739. doi: 10.3390/nano13040739 (PMC9960900; doi:10.3390/nano13040739)
Supplement: Supplementary file 1 [file nanomaterials-13-00739-s001.zip › nanomaterials-2194908-supplementary.pdf]

SUPPLEMENTARY INFORMATION

Engineering multicolor radiative centers in hBN flakes by varying the electron beam irradiation parameters

F Bianco[1], E. Corte[2], S. Ditalia Tchernij[2], J. Forneris[2], F. Fabbri[1]

[1] NEST Laboratory, Istituto Nanoscienze-CNR and Scuola Normale Superiore, Piazza San Silvestro 12, I-56127 Pisa, Italy.

[2] Physics Dept., University of Torino, and Istituto Nazionale di Fisica Nucleare Sez. Torino, via P. Giuria 11, 10125 Torino, Italy.

Figure S1 Atomic Force miscopy analysis of the hBN flake.

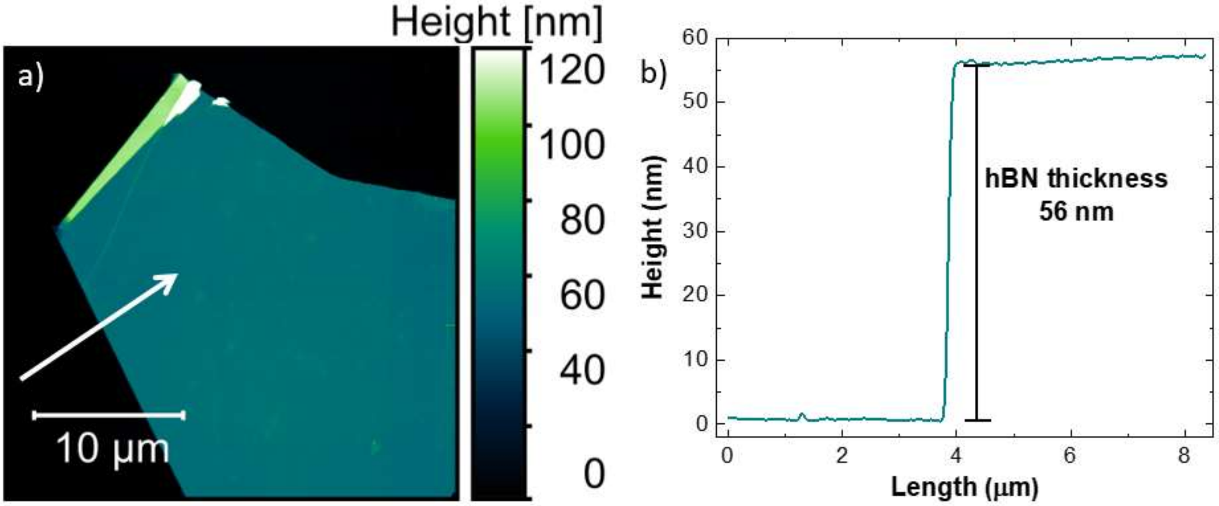

Figure S1: (a) height map of a representative flake employed in this work. The white arrow indicated where the line profile is obtained. AFM measurements are performed using a Dimension Icon AFM (Bruker) operating in Peak Force mode and using ScanAsyst air probe. b) Height line profile obtained, revealing the thickness of the hBN flake, namely 56 nm.

Figure S2 Optical images of the hBN flakes employed in the irradiation processes.

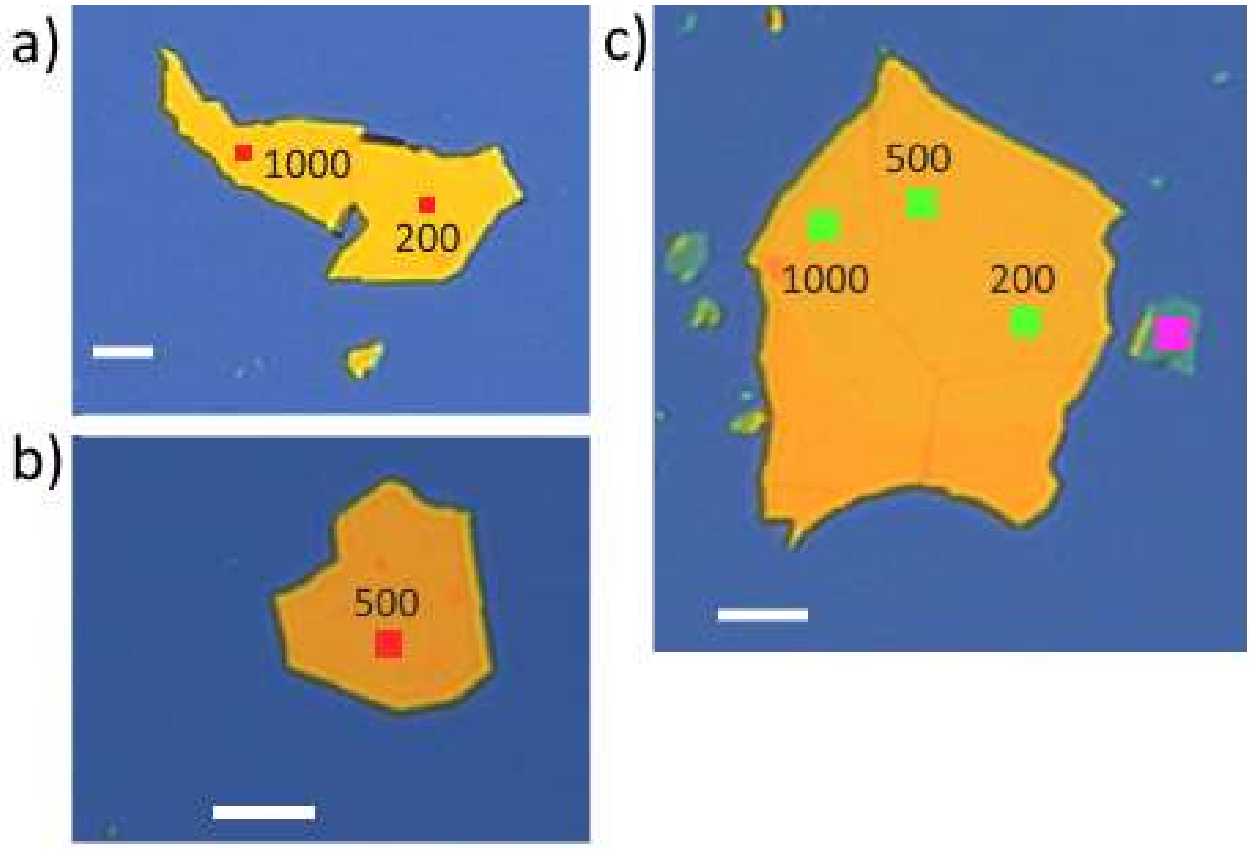

Figure S2: Fig. S2a and S2b show the flakes used in the 10 keV irradiation with a 200 nm, 1000 nm (panel a) and 500 nm (panel b). The red grids indicated the 10 keV irradiated area, while the 15 keV irradiated areas (Fig. S2c) are indicated in green. It is worth noting that in latter case, all the spacing experiments are carried out on the same flake. The purple grid localizes the sacrificial area for electron beam induced carbon deposition prior to the irradiation experiments (Fig. S2c). In all panels, the scale bar is 10 μm.

Figure S3 Monte Carlo simulation of the primary electron beam interaction with the sample.

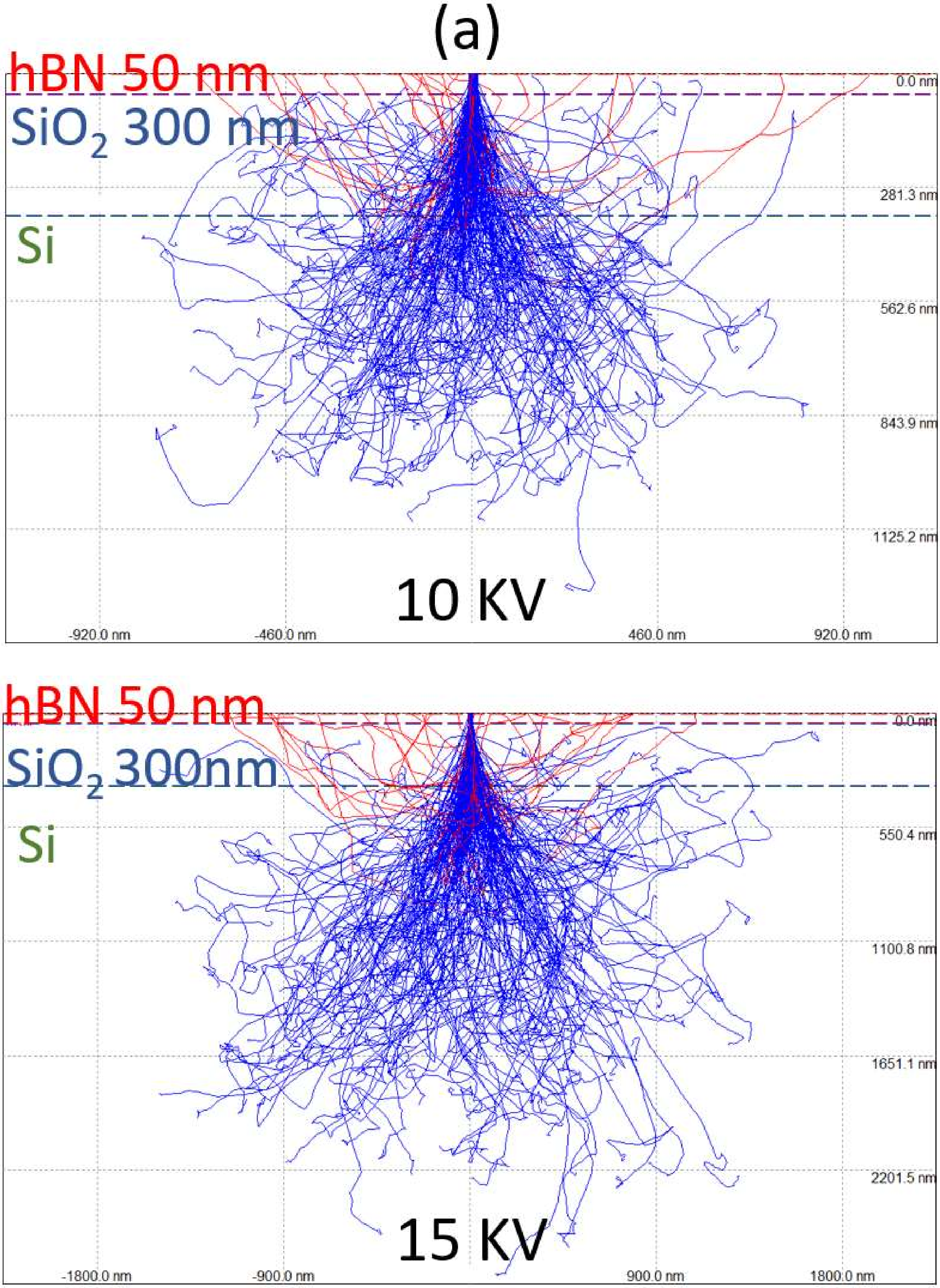

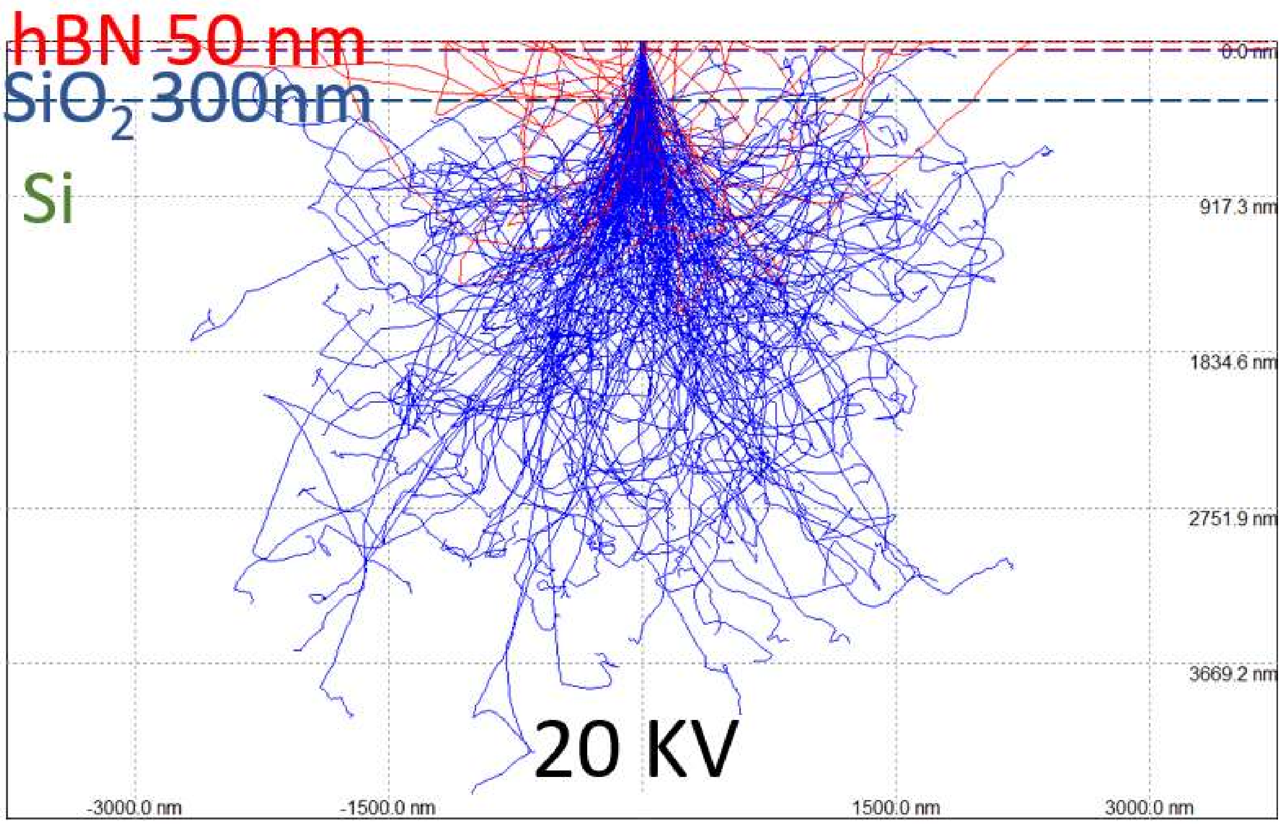
hBN 50 nm
SiO_2 300nm
Si
0.0 nm
917.3 nm
1834.6 nm
2751.9 nm
3669.2 nm
20 KV
-3000.0 nm
-1500.0 nm
1500.0 nm
3000.0 nm

(b)

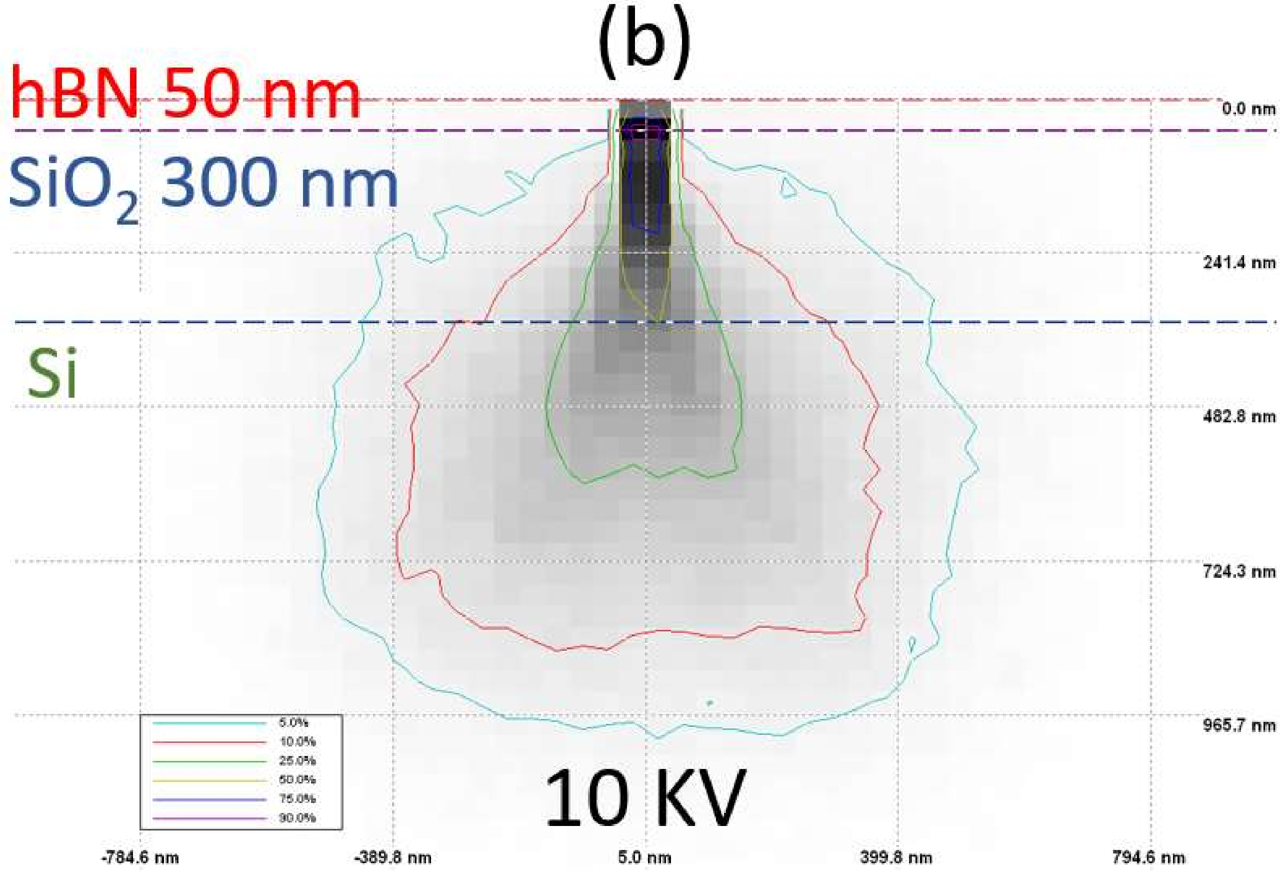
hBN 50 nm
SiO_2 300 nm
Si
0.0 nm
241.4 nm
482.8 nm
724.3 nm
965.7 nm
5.0%
10.0%
25.0%
50.0%
75.0%
90.0%
10 KV
-784.6 nm
-389.8 nm
5.0 nm
399.8 nm
794.6 nm

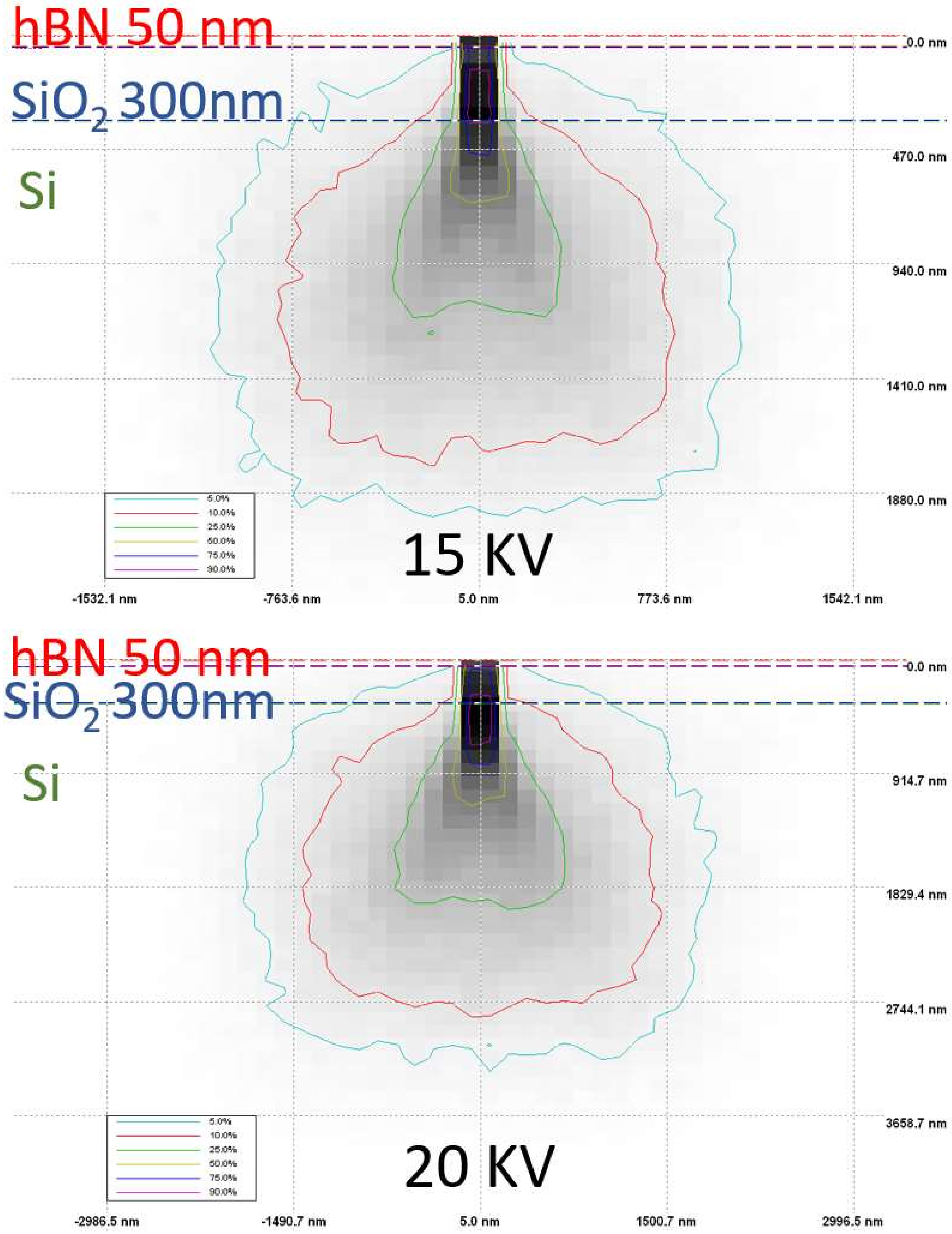

Figure S3: Montecarlo simulation of the electron trajectories (panel a) and for the energy release maps (b) for increasing electron beam energy.

Figure S4 Photoluminescence spectra of electron irradiated bare SiO_2/Si substrate at different electron beam energy.

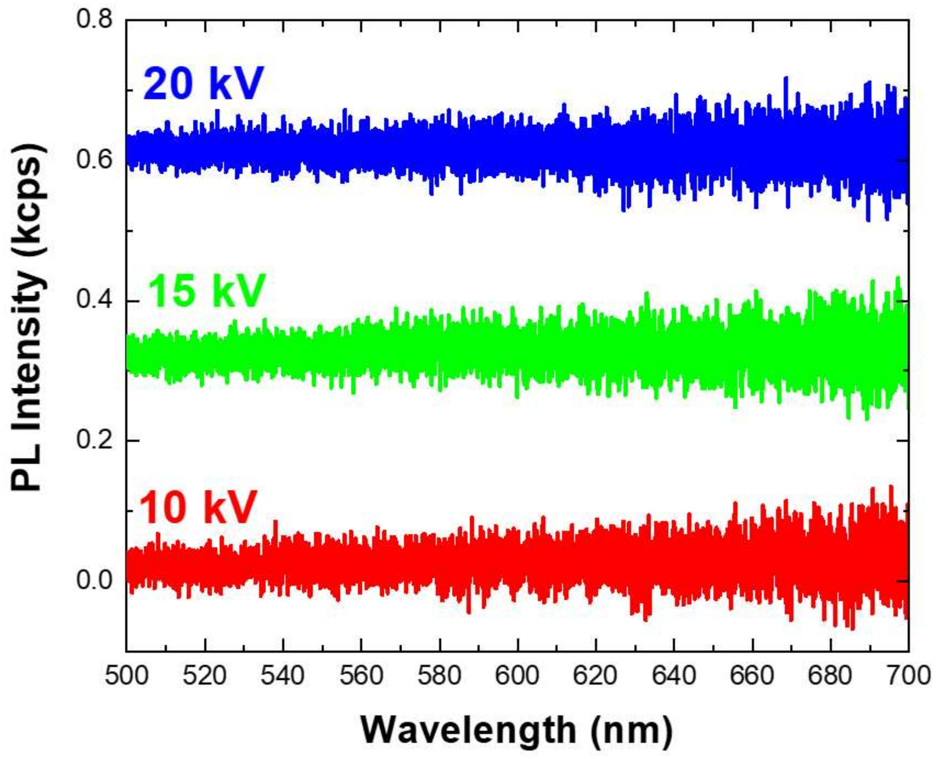

Figure S4: PL spectra of the SiO_2/Si substrate irradiated at different electron beam energy: 10 keV red line, 15 keV green line, 20 keV blue line. Such analysis reveals that the electron beam irradiation does not cause any radiative recombination originated in the SiO_2 layer.

Figure S5 Undesired electron beam induced carbon deposition procedure.

Electron beam carbon deposition was carried out by irradiating a 2 μm x 2 μm area of a thin hBN flake (about 5-10 nm). The irradiation parameters are: kinetic energy of 15 keV, beam current of 0.6 nA, dwell time of 6 s and step size of 100 nm. This allows us the deposition of the contaminant carbon for about 40 minutes. These data are representative for all the irradiation of sacrificial areas carried out at different electron beam energy. Moreover, the flake thickness is chosen for a better localization during the Raman characterization.

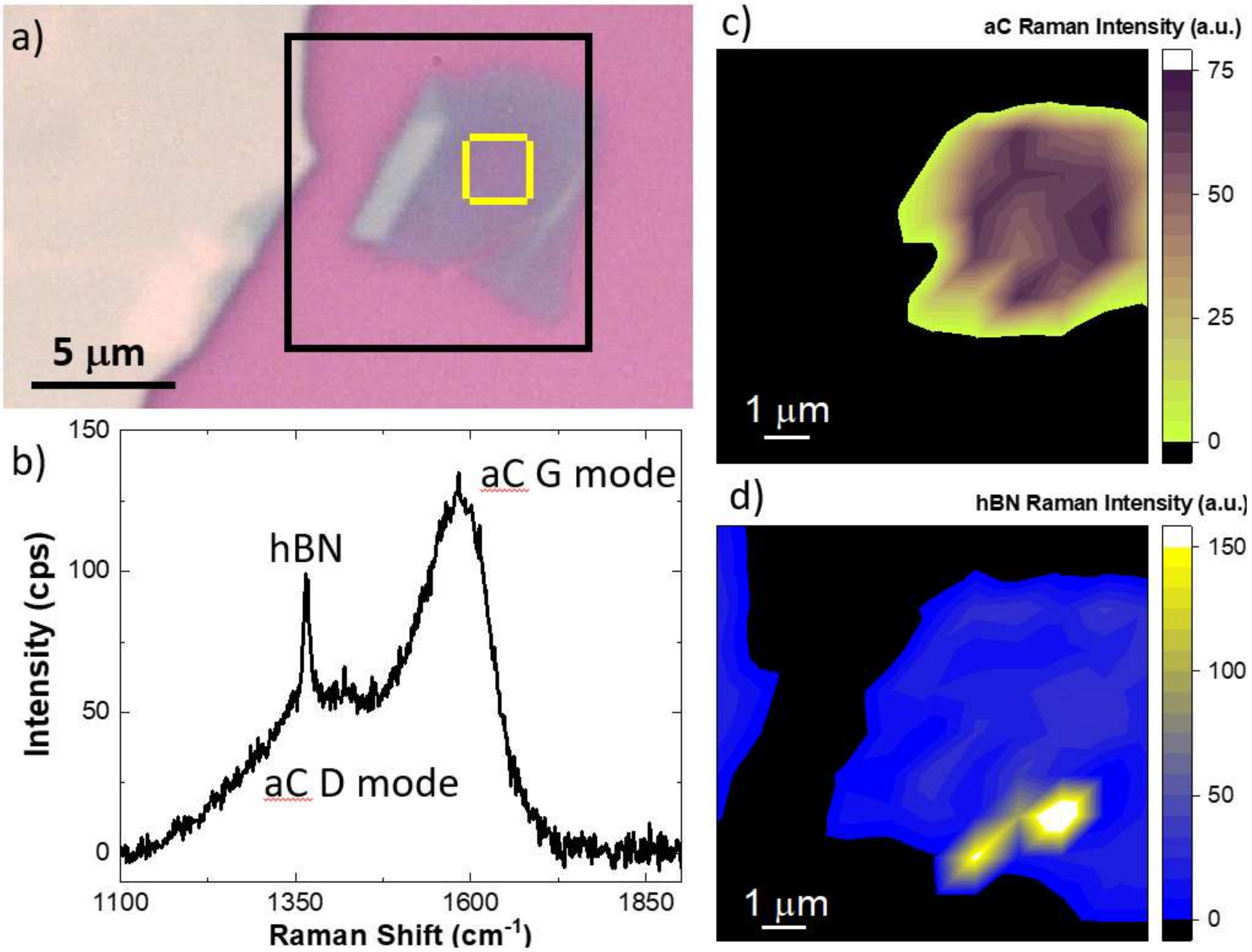

Figure S5: a) optical image of the sacrificial area for the electron beam carbon deposition, carried out prior to the irradiation experiments, in order to get rid of carbon contaminants in the SEM chamber. The black frame indicates the area of the Raman mapping, while the yellow frame shows the irradiated area. b) Raman spectrum, demonstrating the presence of amorphous carbon, where the D peak (1400 cm^{-1}) is superimposed to the hBN sharp mode. The main peak is the G mode (1587 cm^{-1}) attributed to amorphous carbon. c) Raman intensity map of the G mode of amorphous carbon. d) Raman intensity map of the hBN mode.

Figure S6 Optical micrograph of the irradiated areas

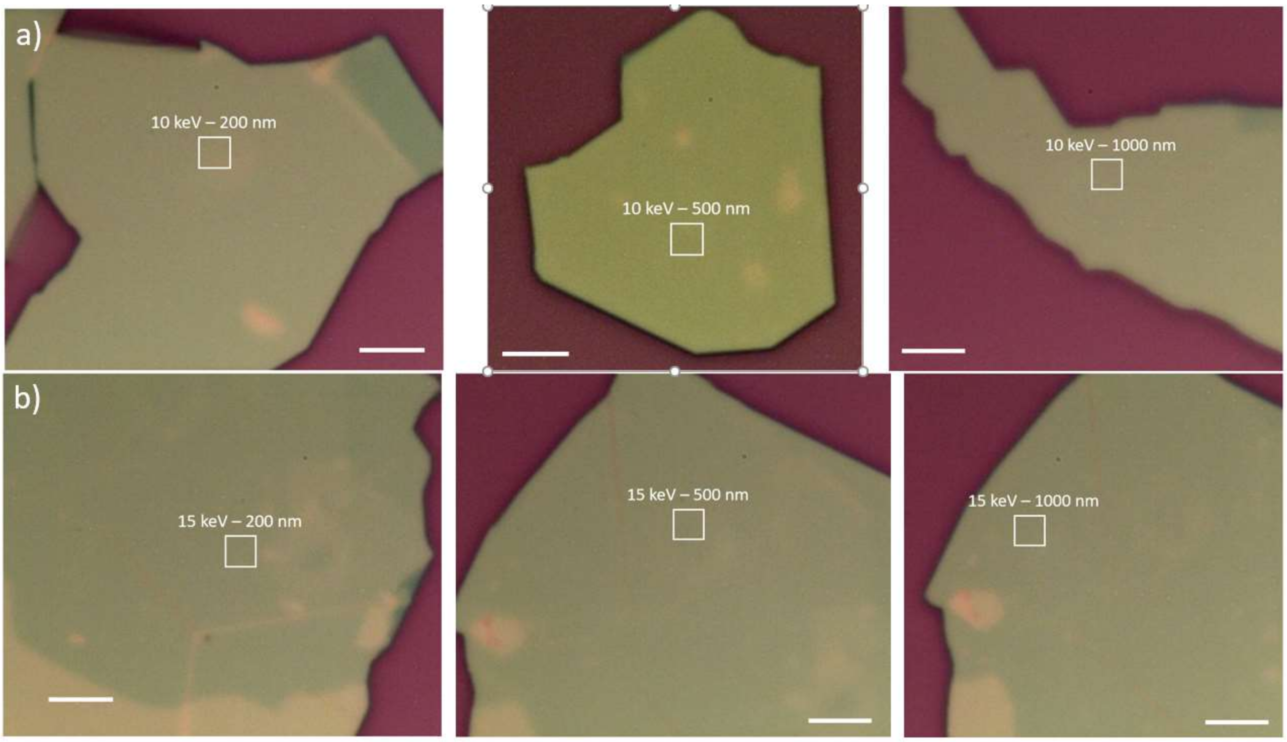

Figure S6: Optical micrograph of the hBN flakes after the irradiation process. The scale bar is 5 μm for all the images. The optical micrographs of the irradiate areas do not show any change of the local optical contrast compared to the surrounding, ruling out any possible effect of beam induced chemical etching of the hBN flake.

Figure S7 Raman mapping of the 15 keV irradiated areas with increasing pattern spacing

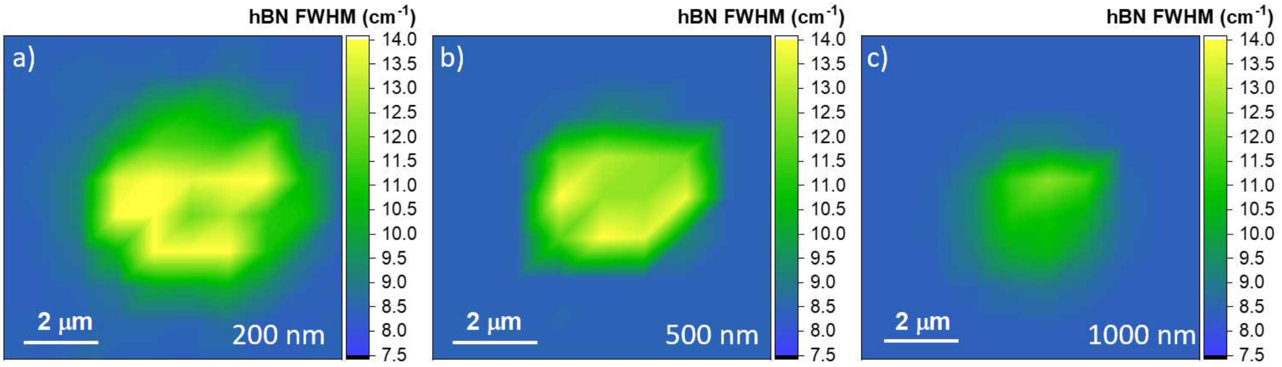

Figure S7: Mapping of the FWHM of the hBN Raman mode in the 15 keV irradiated areas with 200 nm pattern spacing a), 500 nm b) and 1000 nm c). In all the irradiated areas, we report a broadening of the FWHM of the hBN Raman mode.

Figure S8 Raman spectroscopy of the 15 keV irradiated areas with increasing pattern spacing

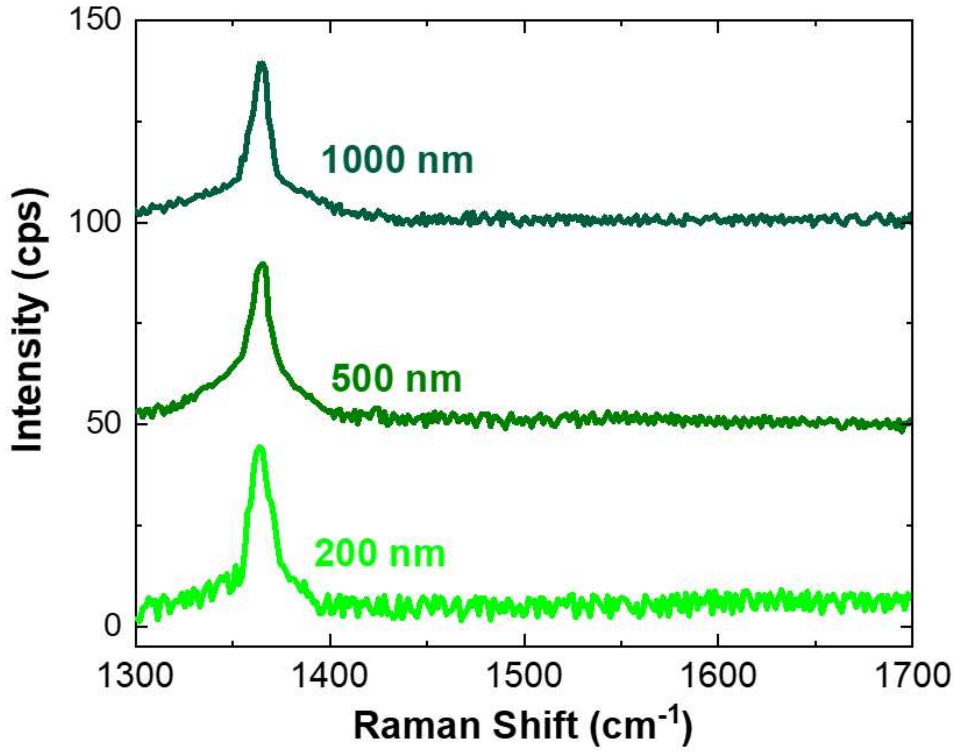

Figure S8: Raman spectra of 15 keV irradiated hBN flakes with increasing pattern spacing. All the spectra present a broadening of the hBN Raman mode and there is a clear indication of the absence of any Raman modes related to the deposition of amorphous carbon. The broadening is evaluated Fig. S6

Figure S9 PL Power dependence fitting.

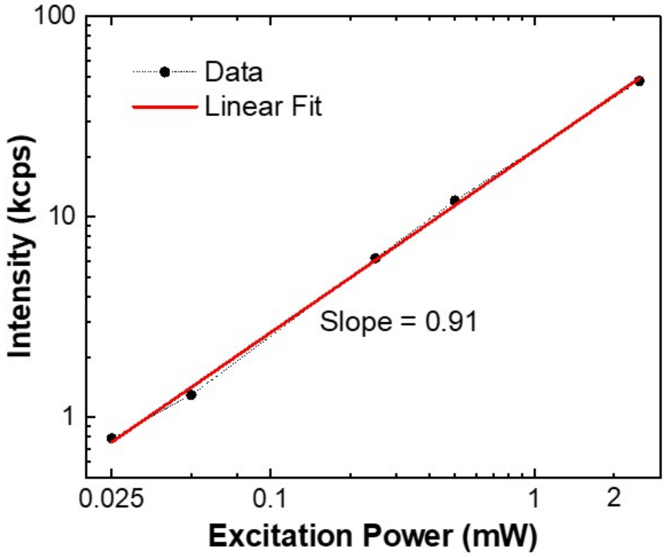

Figure S9: double log plot of the PL peak intensity versus the excitation power (the same data reported in Fig. 5) shows a clear linear behavior, with a slope of 0.91. This slop value is an indication of an excitonic emission bound to an isoelectronic trap.
